# A Designed Experiments Approach to Optimizing MALDI-TOF MS Spectrum Processing Parameters Enhances Detection of Antibiotic Resistance in *Campylobacter jejuni*

**DOI:** 10.3389/fmicb.2016.00818

**Published:** 2016-05-31

**Authors:** Christian Penny, Beau Grothendick, Lin Zhang, Connie M. Borror, Duane Barbano, Angela J. Cornelius, Brent J. Gilpin, Clifton K. Fagerquist, William J. Zaragoza, Michele T. Jay-Russell, Albert J. Lastovica, Catherine Ragimbeau, Henry-Michel Cauchie, Todd R. Sandrin

**Affiliations:** ^1^Environmental Research and Innovation Department, Luxembourg Institute of Science and TechnologyEsch-sur-Alzette, Luxembourg; ^2^School of Mathematical and Natural Sciences, Arizona State University, PhoenixAZ, USA; ^3^Institute of Environmental Science and ResearchChristchurch, New Zealand; ^4^Agricultural Research Service, United States Department of Agriculture, AlbanyCA, USA; ^5^Western Center for Food Safety, University of California at Davis, DavisCA, USA; ^6^Department of Biotechnology, University of the Western CapeBellville, South Africa; ^7^Epidemiological Surveillance of Infectious Diseases, Laboratoire National de SantéDudelange, Luxembourg

**Keywords:** MALDI-TOF MS, *Campylobacter jejuni*, antimicrobial resistance, antibiotic resistance, designed experiments, spectrum processing

## Abstract

MALDI-TOF MS has been utilized as a reliable and rapid tool for microbial fingerprinting at the genus and species levels. Recently, there has been keen interest in using MALDI-TOF MS beyond the genus and species levels to rapidly identify antibiotic resistant strains of bacteria. The purpose of this study was to enhance strain level resolution for *Campylobacter jejuni* through the optimization of spectrum processing parameters using a series of designed experiments. A collection of 172 strains of *C. jejuni* were collected from Luxembourg, New Zealand, North America, and South Africa, consisting of four groups of antibiotic resistant isolates. The groups included: (1) 65 strains resistant to cefoperazone (2) 26 resistant to cefoperazone and beta-lactams (3) 5 strains resistant to cefoperazone, beta-lactams, and tetracycline, and (4) 76 strains resistant to cefoperazone, teicoplanin, amphotericin, B and cephalothin. Initially, a model set of 16 strains (three biological replicates and three technical replicates per isolate, yielding a total of 144 spectra) of *C. jejuni* was subjected to each designed experiment to enhance detection of antibiotic resistance. The most optimal parameters were applied to the larger collection of 172 isolates (two biological replicates and three technical replicates per isolate, yielding a total of 1,031 spectra). We observed an increase in antibiotic resistance detection whenever either a curve based similarity coefficient (Pearson or ranked Pearson) was applied rather than a peak based (Dice) and/or the optimized preprocessing parameters were applied. Increases in antimicrobial resistance detection were scored using the jackknife maximum similarity technique following cluster analysis. From the first four groups of antibiotic resistant isolates, the optimized preprocessing parameters increased detection respective to the aforementioned groups by: (1) 5% (2) 9% (3) 10%, and (4) 2%. An additional second categorization was created from the collection consisting of 31 strains resistant to beta-lactams and 141 strains sensitive to beta-lactams. Applying optimal preprocessing parameters, beta-lactam resistance detection was increased by 34%. These results suggest that spectrum processing parameters, which are rarely optimized or adjusted, affect the performance of MALDI-TOF MS-based detection of antibiotic resistance and can be fine-tuned to enhance screening performance.

## Introduction

MALDI-TOF MS has revolutionized the field of molecular microbial diagnostics in recent years (Sauer and Kliem, 2010; Welker, 2011; Welker and Moore, 2011; Kliem and Sauer, 2012). This approach has been implemented in biomedical, veterinary and environmental routine procedures for bacterial identification at the genus, species, and sometimes at the subspecies level (e.g., Wieser et al., 2011; Croxatto et al., 2012; Koubek et al., 2012; Lartigue, 2013; Sandrin et al., 2013; Randall et al., 2015). While microbial identification using MALDI-TOF MS is rapid and reliable, the taxonomic resolution obtained from the mass spectra is not always sufficient, or the bioinformatics software pipeline is not optimized or adapted, for typing the candidate bacteria below the species level (Sandrin et al., 2013; Zhang et al., 2014). However, some studies have shown that categorization of strains of bacteria with respect to their membership in nucleic acid-based subgroups, pathogenicity traits or antimicrobial resistance (AMR) identification is indeed feasible, but depends on the level of variability inside a given taxon as well as on the precise identification of characteristic biomarkers using bioinformatics tools (Sandrin et al., 2013). Fine-tuning of mass spectrum analysis is evidently mandatory.

Rigorous analysis of spectra has permitted successful detection of AMR (e.g., Hrabák et al., 2013; Kostrzewa et al., 2013; Pulido et al., 2013), but further development is needed to render MALDI-based approaches a more routine, reliable, and effective alternative to traditional methods. As has been shown in several MALDI applications to discriminate bacterial strains (Mitchell et al., 2015), high reproducibility is required for reliable AMR detection. Although many portions of the MALDI-TOF MS workflow such as sample preparation and data acquisition have been optimized with regard to spectrum reproducibility and method performance (e.g., Freiwald and Sauer, 2009; Goldstein et al., 2013), no standardization for mass spectrum processing parameters has been proposed. Processing parameters are used to calculate, define, and resolve acquired spectra into interpretable data. Baseline subtraction, a common processing parameter, establishes a baseline from the spectrum, leaving a clearer picture of the remaining peaks. Another processing parameter, smoothing, reduces background noise, and increases signal-to-noise ratio. The manner in which the data are translated by processing parameters may affect the ability of MALDI-TOF MS-based fingerprinting to detect antibiotic resistance. In addition, different software packages that use distinct spectrum processing workflows and parameters are often used. Many of these programs do not offer the ability to alter and optimize spectrum processing parameters. Such optimization, though, may be necessary to enhance method performance, particularly with regard to resolving strain-level differences, such as AMR.

The issues regarding the ever-increasing bacterial resistance to large categories of antimicrobial compounds are particularly of public health concern for the world’s leading bacterial gastro-enteritis agent *Campylobacter* (World Health Organization [WHO], 2013). The species *Campylobacter jejuni* is recognized as the major food- and waterborne pathogen inside this taxon, and is a major threat to public health (Kaakoush et al., 2015; Wagenaar et al., 2015). AMR in *Campylobacter* is steadily increasing (e.g., Luangtongkum et al., 2009; Ge et al., 2013; Iovine, 2013; Wieczorek and Osek, 2013). Of particular concern is also the increasing incidence of AMR of *Campylobacter* spp. other than *C. jejuni*, whose disease potential is not fully appreciated at present (Lastovica, 2006; Lastovica et al., 2014). *Campylobacter* easily undergoes DNA transformation by foreign exogenous DNA resulting in many different antibiotic-resistant strains (Bae et al., 2014). Also, antibiotic resistance mutations in *C. jejuni* continue to develop (Iovine, 2013). For example, a single mutation in the gyrase subunit A (*gyr*A) gene, resulting in an amino acid substitution, is sufficient for conferring resistance to quinolones (Wang et al., 1993; Payot et al., 2006).

In diagnostic and clinical microbiology, as well as epidemiological surveillance, the need for implementation of early and precise information retrieval concerning AMR has been raised (Laxminarayan et al., 2013). This could greatly improve treatment of infectious diseases and help limit the spread of multiple resistant strains of harmful bacteria. In this context, the evolution toward microbial characterization, and more specifically AMR prediction through whole-genome sequencing (WGS) has been described in recent years including the identification of AMR-specific signatures in *Campylobacter* (e.g., Didelot et al., 2012; Zhao et al., 2016). But also, the potential of various MALDI-TOF MS applications for the prediction of AMR mechanisms has been identified (Hrabák et al., 2013; Kostrzewa et al., 2013). Most importantly, the use of mass spectrometry toolkits for the diagnosis of AMR in *Campylobacter* is emerging (Wieser et al., 2011; Lartigue, 2013; Schubert and Kostrzewa, 2015), and a new microbial typing method relying on mass spectrometry-based phyloproteomics (MSPP), permitting biomarker, and genetic features characterization in *Campylobacter*, has recently been published (Zautner et al., 2015). Still, the MALDI-TOF MS-based workflow remains in the need of optimization and simplification of robust, reliable and reproducible workflows, especially regarding data handling after automated routine acquisition of mass spectra.

Consequently, the overarching objective of this study was to determine whether MALDI-TOF mass spectrum processing parameters could be optimized to enhance the detection of antibiotic resistance in clinically relevant environmental, animal, and human isolates of *C. jejuni*. Therefore, 172 isolates of *C. jejuni* were collected from four continents, some of which share antibiotic resistances within four different groups (**Table 1**). Special emphasis was put on *C. jejuni* resistance to beta-lactams, as this antibiotics group is considered among the most important and widespread treatment with resistance issues (Wieser et al., 2011; Lartigue, 2013; Schubert and Kostrzewa, 2015). A designed experiments approach was employed (Zhang et al., 2014), in which spectrum processing parameters were varied to optimize detection of AMR. Translation of the genetic and phenotypic characteristics of *C. jejuni* might identify useful and straightforward information collection in a global One Health context (Maloy and Atlas, 2014). Our results suggest that a designed experiments approach allows optimization of mass spectrum analysis and enhances detection of AMR in *C. jejuni*.

**Table 1:** Characteristics of the *Campylobacter jejuni* isolates used in this study.

*C. jejuni* isolate collection	
Number of strains	172
Geographic origin	Luxembourg, New Zealand, South Africa, USA
Sources	Alpaca, bovine, chicken, feral swine, goat, goose, human, milk product, ovine, raccoon, surface water, turkey, vole, wastewater, wildfowl
Antibiotic resistance profiles (phenotypically or genotypically confirmed resistances)	Group 1 (65 isolates): Cefoperazone	
	Group 2 (26 isolates): Beta-lactams, cefoperazone
	Group 3 (5 isolates): Beta-lactams, cefoperazone, tetracycline
	Group 4 (76 isolates): Cefoperazone, teicoplanin, amphotericin B, cephalotin


## Materials and Methods

### *Campylobacter jejuni* Strains and Culture Conditions

A collection of 16 *C. jejuni* isolates were used as a model system for the designed experiments and a total of 172 *C. jejuni* strains from various geographical and animal host origins were used in application of the model spectrum processing parameters (**Table 1**).

Antibiotic resistance profiles were established by a non-exhaustive series of phenotypic and genomic attribute tests of the *C. jejuni* isolate collection, depending on specific culture media used and availability of whole genome sequence data (WGS) of the strains. Genomics-based AMR potential of part of the collection was obtained through screening using the ResFinder bioinformatics platform^1^ (Zankari et al., 2012).

For each strain, chocolate agar plates (Remel Microbiology Products, Lenexa, KS, USA) were inoculated with stock suspensions stored at -80°C in FBP medium (Gorman and Adley, 2004), and incubated for 40 ± 4 h at 42°C under microaerobic conditions in gastight jars (2.5 L, Remel) using CampyGen 2.5 L gaspacks (Remel). For biological replicates, the same stock suspension was streaked onto two to three separate chocolate agar plates on different days.

### Sample Preparation

A previously described protein extraction sample preparation method was employed with minor modifications (Freiwald and Sauer, 2009). Briefly, cells from 40 ± 4 h cultures were pelleted by centrifugation (17,000 × *g* for 5 min) and washed with sterile double-distilled water (ddH_2_O) (Millipore Corp.; Bedford, MA, USA). Cells were re-suspended in sterile ddH_2_O, and the cell density of each suspension was adjusted to 0.8 ± 0.1 OD_600_. Each 1 mL cell suspension was pelleted by centrifugation (17,000 × *g* for 5 min), and the supernatant was carefully removed. Cell pellets were inactivated by resuspension in 800 μL of absolute ethanol and 300 μL sterile ddH_2_O. Inactivation was verified by streaking a loopful of the inactivated suspension onto chocolate agar and verification of absence of colony formation after 72 h of incubation under the conditions described above. Each sample was centrifuged (17,000 × *g* for 5 min), and the resulting supernatant was discarded. A washing step with 1 mL ddH_2_O was performed on each cell pellet. Twenty-five microliters of 70% formic acid (Sigma–Aldrich, St. Louis, MO, USA) and 25 μL acetonitrile (Alfa Aesar, Ward Hill, MA, USA) were mixed with the pellet by vortexing thoroughly. Each sample was centrifuged (17,000 × *g* for 5 min), and the supernatant containing the protein extract was transferred into a sterile 1.5 mL microcentrifuge tube. Protein extract (1.0 μL) was pipetted onto a polished steel 96-well MALDI target plate (Bruker Daltonics, Billerica, MA, USA) and allowed to air-dry for 10 min. Samples were spotted onto predetermined, randomly distributed locations on the target plate. After the sample had dried, it was overlaid with 1.0 μL of α-cyano-4-hydroxycinnamic acid (ACROS, Fair Lawn, NJ, USA) matrix prepared in 50% acetonitrile and supplemented with 2.5% trifluoroacetic acid (ACROS, Fair Lawn, NJ, USA). Each isolate was spotted in three technical replicates per biological replicate.

### MALDI-TOF MS Data Acquisition

MALDI-TOF MS analyses were performed using a Bruker Microflex LRF MALDI-TOF mass spectrometer (Bruker Daltonics) equipped with a nitrogen laser (λ = 337 nm) under the control of FlexControl software (v. 3; Bruker Daltonics). Each spectrum was obtained in a linear, positive ion mode and calibrated externally using ACTH (1–17) (2094.427 Da), ACTH (18–39) (2466.681 Da), insulin oxidized B (3494.651 Da), insulin (5734.518 Da), cytochrome C (12360.974 Da), and myoglobin (16952.306 Da). Data acquisition was performed automatically in steps of 100 shots for a total of 500 shots. Laser power was set to the necessary minimum power for ionization of selected samples before starting the analyses. The signal-to-noise threshold was set at two, the minimum intensity threshold at 100, and the maximum number of peaks to 500. Peak width was set at 10 m/z and a height of 80%.

### Spectrum Cluster Analysis

Mass spectra were exported from FlexAnalysis (version 3.0; Bruker Daltonics) as .txt files and imported into BioNumerics (version 7.1; Applied Maths, Sint-Martens-Latem, Belgium). Spectra were initially pre-processed using the default program settings (Baseline Subtraction, 1; Rolling Disk, 200). For cluster analysis, spectra were compared pairwise using the Pearson correlation coefficient. The Dice similarity coefficient, in which lists of peaks containing only binary values (present or absent) were generated from spectra, was also evaluated. A dendrogram was generated using the unweighted pair group method with an arithmetic mean (UPGMA) algorithm. Multidimensional scaling (MDS) analysis was performed as previously described to visualize the similarity between spectra (Goldstein et al., 2013). Jackknife analysis was performed as described previously using maximum similarities to quantify rates of correct classification with regard to AMR (Giebel et al., 2008).

### Processing Steps and Settings Selection: Designed Experiments

Processing steps chosen as factors were based on those commonly cited in literature and an initial descriptive analysis of their effects on the response (jackknife score). These were found to be important in prior work in our lab, because of their ability to affect number of peaks and spectrum quality. The steps chosen were baseline subtraction, smoothing, and similarity coefficient. The levels, or different methods of baseline subtraction, smoothing, and similarity coefficient, were considered categorical variables, and each step was considered as a categorical variable (**Table 2A**). The highest scoring levels from each category were then selected and further optimized based on their numerical settings (**Table 2B**).

**Table 2A:** (A) Factors and levels used in the designed experiments for processing MALDI-TOF MS spectra of the *C. jejuni* collection.

Processing steps (factors)	Processing options (levels)
Baseline Subtraction 1: Binned Baseline	Binned Baseline, Monotone Minimum, Moving Bar, Rolling Disk
Smoothing: Kaiser Window	Gaussian, Kaiser window, Moving Average, Savitzky–Golay
Baseline Subtraction 2: Moving Bar	Binned Baseline, Monotone Minimum, Moving Bar, Rolling Disk
Similarity Coefficient	Dice, Pearson, Ranked Pearson


**Table 2B:** (B) Factors and levels used in the designed experiments approach with center points for highest scoring in predicted settings.

Processing options (factors)	Processing settings (levels)
Binned Baseline	Bin Size of 4, 77, 150
Kaiser Window	Window Size of 1, 21, or 40
Moving Bar	Bar Width of 3, 102, or 201


### Statistical Analyses

Each of the two/three biological replicates contained three technical replicates. Each set of biological replicates was composed of sixty runs of calculations with varying processing step settings. All 180 experimental runs were carried out in 3 days in random order, and grouped into blocks by both day and biological replicate. Blocking, or grouping together, based on the aforementioned variables, helped to reduce sources of variability and increase precision (Montgomery, 2012). The datasets were subjected to analysis of main effects, interaction of factors, best, and worst combination of settings, significant factors affecting spectrum qualities, and *post hoc* tests on factors. Main effects and interactions of factors on reproducibility were analyzed based on analysis of variance (ANOVA) and *t*-tests using a 5% level of significance. *Post hoc* tests were performed using Tukey’s test (Minitab Inc., PA, USA).

### Parameter Optimization

An optimized setting for preprocessing parameters and similarity coefficient was determined using ANOVA. The optimized setting was applied to each dataset. The jackknife score (response) from each dataset was reported using either the default settings or optimized preprocessing settings with combinations of three different similarity coefficients: Dice, Pearson, and ranked Pearson. Jackknife analysis was used as described previously (Goldstein et al., 2013) to evaluate the extent to which MALDI-TOF MS profiles were assigned to particular AMR categories. Differences in spectrum quality and jackknife scores before and after optimization were identified using *t*-tests with a 5% level of significance. A second dendrogram and MDS were visualized following optimization. Both optimal and default preprocessing settings were applied to larger sets of *C. jejuni*. In addition, varying similarity coefficients, and jackknife scores were used to measure the effectiveness of each processing parameter combination.

## Results

A total of 172 different strains of *C. jejuni* were analyzed for this study. The sample collection was representative of diverse environments and hosts from four continents (**Table 1**). The MALDI-TOF MS profile of each strain was constituted using two sets of biological replicates, and each biological replicate was analyzed using three technical replicates (LX-32 is an exception due to the corruption of a single spectrum in biological replicate B2). Thus, the total number of spectra generated for this analysis was 1,031 spectra.

**FIGURE 1:**
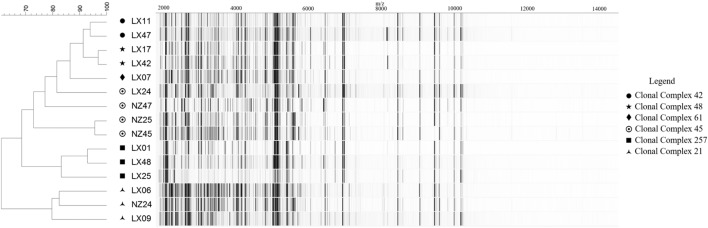
**MALDI-TOF MS spectra-based clustering of the *Campylobacter jejuni* reference strain collection conforming to the genetic fingerprints.** Spectra are represented as pseudo-gels. Calculations and clustering were obtained using default settings in the BioNumerics 7.1. Genomic profiles are expressed as Clonal Complexes (CC) obtained through the standardized Multi-Locus Sequence Typing (MLST) method of Jolley and Maiden (2010).

Within the strain collection, a model set of 16 *C. jejuni* isolates was used for optimizing spectrum preprocessing methods at levels below the species. Organisms for the model set were selected based on (i) their genetic fingerprints, considering six major Clonal Complexes (CCs) obtained through MLST analysis and represented by one to four isolates each (with varying host sources and origins), and (ii) their respective MALDI-TOF MS spectrum profiles with regard to shared base peaks and varying degrees of peak intensity (**Figure 1**). For the model set, we utilized three biological replicates with three technical replicates each and generated a total of 144 spectra.

Relying on the example of the model set of isolates (excluding isolate LX-41 whose CC affiliation was not confirmed), it could be demonstrated that MALDI-TOF MS-based clustering of *C. jejuni*, based on peak mass and intensity ranges within the spectrum, was concordant with the genotype profiles expressed as CCs (**Figure 1**). All strains, except two isolates from CC-45, clustered together with their genetically closest neighbors from the same CC. Genetic diversity information on *Campylobacter* isolates could therefore be transcribed through MALDI-TOF MS fingerprinting profiles. This highlighted the potential of mass spectrometry for clonality prediction inside bacterial taxa. Then, characteristic traits such as AMR can individually be screened within each isolate, following optimization in the bioinformatics workflow for spectrum processing and MS-based clustering and typing.

In this context, we subsequently analyzed our model set of *C. jejuni* using the initial designed experiments approach for enhancing AMR detection and strain clustering into their respective groups of AMR (**Table 1**). The parameters for optimization were split into factors and then their individual components were referred to as levels (**Tables 2A,B**). The most optimal combination of the four parameters was found to be the following: (i) Binned Baseline (Bin Size of 77), (ii) Kaiser Window (Window Size of 33), (iii) Moving Bar (Bar Width of 129), and (iv) ranked Pearson similarity coefficient.

Following the model set analysis, these optimized parameters were applied to all spectra from the collection. Increases in rates of correct classification with regard to AMR, when switching from default settings to optimized settings, were observed (**Table 3**). Considering all four groupings of antibiotic-resistant *C. jejuni*, we observed a 5% increase in group 1, a 9% increase in group 2, a 10% increase in group 3, and finally, a 2% increase in group 4. Overall, use of optimized settings yielded a significant 7% increase (*t*-test, *p* = 0.05) in detection of AMR when compared to use of default settings. In each of the antibiotic resistance groups, the Pearson correlation coefficient outperformed the Dice similarity coefficient (*p* = 0.0002). In only one instance was there a decrease in the rate of correct classification when using optimized settings. When the settings for group 3 were swapped from default to optimal with the Pearson coefficient, we observed a decrease from 100 to 97% (**Table 3**).

**Table 3:** Effect of MALDI-TOF MS spectrum processing parameters on AMR detection in strains of *C. jejuni.*

Antibiotic resistance group	Preprocessing	Similarity coefficient	Score
1	Default	Dice	89 ± 5
2	Default	Dice	87 ± 6
3	Default	Dice	73 ± 9
4	Default	Dice	93 ± 3
1	Default	Pearson	98 ± 1
2	Default	Pearson	97 ± 3
3	Default	Pearson	100 ± 5
4	Default	Pearson	100 ± 1
1	Default	Ranked	85 ± 14
2	Default	Ranked	73 ± 6
3	Default	Ranked	73 ± 28
4	Default	Ranked	98 ± 2
1	Optimized	Dice	92 ± 5
2	Optimized	Dice	88 ± 7
3	Optimized	Dice	80 ± 9
4	Optimized	Dice	96 ± 3
1	Optimized	Pearson	99 ± 0
2	Optimized	Pearson	98 ± 3
3	Optimized	Pearson	97 ± 5
4	Optimized	Pearson	100 ± 0
1	Optimized	Ranked	97 ± 3
2	Optimized	Ranked	97 ± 4
3	Optimized	Ranked	100 ± 0
4	Optimized	Ranked	100 ± 1


*Scores represent mean (±SD) jackknife values calculated using maximum similarities.*

**FIGURE 2:**
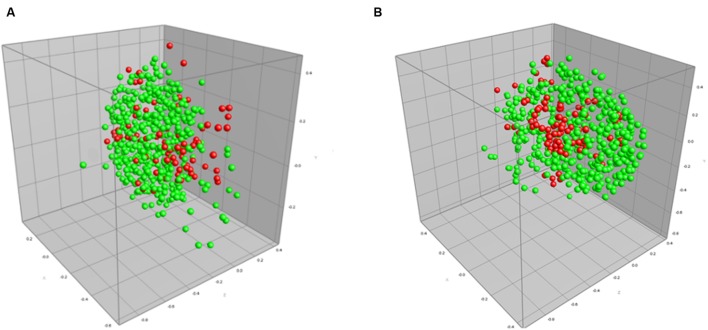
**Multidimensional scaling (MDS) representation of MALDI-TOF mass spectra of 172 isolates (516 spectra) of beta-lactam resistant (red) and sensitive (green) strains of *C. jejuni* using **(A)** default spectrum processing settings and **(B)** optimized processing settings defined in the designed experiments approach in this study.** The ranked Pearson correlation coefficient was used to quantify similarity in both the default and optimized cases.

We also performed a direct comparison between default processing settings and the optimized processing settings when applied to isolates exhibiting beta-lactam resistance (**Figure 2**; **Table 4**). The MDS representing optimized parameters shows tighter grouping compared to the default parameter MDS, thus representing an increase in AMR detection, and here specifically beta-lactam resistance. Use of optimized spectrum processing settings increased the rate of correct classification from 63.5 to 95.7%.

**Table 4:** Optimized spectrum preprocessing parameters enhance beta-lactam-resistance detection in *C. jejuni*.

Preprocessing	Similarity coefficient	Score
Default	Dice	86 ± 5
Default	Pearson	97 ± 3
Default	Ranked	74 ± 18
Optimized	Dice	89 ± 8
Optimized	Pearson	98 ± 2
Optimized	Ranked	98 ± 3


*Scores represent mean (±SD) jackknife values calculated using maximum similarities.*

## Discussion

Workflows to rapidly characterize bacteria using MALDI-TOF MS typically include four components: (i) culturing, (ii) sample preparation, (iii) data acquisition, and (iv) data analysis (Sandrin et al., 2013). Each of the first three components of this common workflow have been shown previously to affect the ability of MALDI-TOF MS to reliably and accurately characterize bacteria at the strain level (Schumaker et al., 2012; Goldstein et al., 2013). Results presented here show clearly that the fourth component of this workflow, data analysis (spectrum processing parameters), affects the ability of MALDI-TOF MS to detect AMR in *C. jejuni*. To our knowledge, this work represents the first report of enhancing MALDI-TOF performance to detect AMR in *C. jejuni* through optimization of spectrum processing parameters.

Designed experiments have been used previously to enhance MALDI-TOF MS-based characterization of bacteria (Zhang et al., 2014). In that work, the third component of the MALDI-TOF MS workflow, data acquisition, was enhanced by systematically adjusting parameters (e.g., threshold base peak, S–N resolution, etc.) in an algorithm commonly used for automated spectrum acquisition. Similar to our work here applying designed experiments to data analysis, Zhang et al. (2014) reported increases in method performance (reproducibility) with optimization afforded by designed experiments. Furthermore, Zhang et al. (2014) reported that optimized data acquisition parameters obtained with one bacterium (*Pseudmonas aeruginosa*) were useful in increasing reproducibility of spectra of other bacteria (*Klebsiella pneumoniae* and *Serratia marcescens*). Further research is warranted to determine whether the optimized settings we identified here to enhance detection of AMR in *C. jejuni*, will enhance detection of AMR in other bacteria. Currently, the necessity for adapting the settings of variables and parameters for each individual microorganism or taxon is rigorously being evaluated, but in that case optimization is facilitated with the use of the designed experiments approach described here.

Following spectrum processing (i.e., baseline subtraction, smoothing, etc.), the similarity of processed spectra is often quantified using previously described similarity coefficients including the Dice similarity coefficient, the Pearson correlation coefficient, or the ranked Pearson correlation coefficient (Dieckmann et al., 2008; Schmidt et al., 2009; Sandrin et al., 2013). Each of these coefficients has been used previously to compare spectra and characterize diverse bacteria using MALDI-TOF MS. Our work here suggests that the Dice correlation coefficient underperforms in comparison to Pearson correlation coefficients, particularly the ranked Pearson correlation coefficient. This is in accordance with our prior work with *Enterococcus* (Giebel et al., 2008), in which we reported the importance of considering peak intensity information, which is included in the Pearson correlation coefficient calculations but not the Dice coefficient. For this reason, future efforts to detect AMR using MALDI-TOF MS should use correlation coefficients that include peak intensity, such as the Pearson correlation coefficient.

Most likely in complement to current tools for *Campylobacter* fingerprinting, MALDI-TOF MS spectra indeed reflect the genetic diversity (Zautner et al., 2013), but more importantly reflect the actual genetic expression profile and AMR potential of the strain candidates upon isolation and culture. The designed experiments approach described here appears in this way as a convenient bioinformatics tool for the optimization of information retrieval from MALDI-TOF MS spectra. BioNumerics currently represents the most versatile and flexible routinely used software package for screening optimal processing variable values and parameters such as described in **Table 2**.

Within the next steps, further AMR profiling of *Campylobacter* based on MALDI-TOF MS should be undertaken, in order to obtain more complete AMR profiles in the future. Using extended bioinformatics and proteomics tools, AMR signature identification is likely to be pursued, e.g., by characterizing specific beta-lactamase biomarker(s) within the resistant *C. jejuni* mass spectra (Sparbier et al., 2012; Kostrzewa et al., 2013). Here, bottom–up and top–down proteomics approaches could be deployed in order to complement and supplement existing tools (e.g., Fagerquist et al., 2005, 2009; Sandrin et al., 2013). Also, further comparisons with AMR prediction through WGS data or MSPP phyloproteomics screening will certainly be fruitful (Zankari et al., 2012; Zautner et al., 2015).

The features and potential of MALDI-TOF MS will continue to contribute to significant scientific and technological advances in the fields of functional characterization and fingerprinting of microorganisms. As shown here, optimized bioinformatics workflows inside MALDI-TOF MS analysis will, among others, allow enhanced AMR detection for improved decision-making and healthcare through implementation in microbial subspecies typing and diagnostics. It would be interesting to adapt the analysis software inside the routine workflow by adding an AMR detection module fed with the optimized parameter settings for data acquisition, processing, and biomarker screening. Constitution of AMR profiles using *Campylobacter*-specific, sensitive and reproducible analysis parameters, such as those set-up here, will greatly add value to limiting the ongoing emergence of multi-drug resistances in *Campylobacter* sp.

## Author Contributions

CP, H-MC and TS designed the study. CP, BG, LZ, CB, DB, and TS were responsible for data acquisition and analysis. CP, AC, BG, AL, MJ-R, CR, CK, and WZ contributed to the *C. jejuni* strain collection set-up and the AMR profiling and genetic fingerprinting of the isolates. All authors contributed to the writing and/or critical reviewing of the manuscript, and have approved the manuscript.

## Conflict of Interest Statement

The authors declare that the research was conducted in the absence of any commercial or financial relationships that could be construed as a potential conflict of interest.

## Footnotes

1 https://cge.cbs.dtu.dk//services/ResFinder/

## References

[B1] Bae J. Oh E. Jeon B. (2014). Enhanced transmission of antibiotic resistance in *Campylobacter jejuni* biofilms by natural transformation. *Antimicrob. Agents Chemother.* 58 7573–7575. 10.1128/AAC.04066-14 25267685 PMC4249540

[B2] Croxatto A. Prod’hom G. Greub G. (2012). Applications of MALDI-TOF mass spectrometry in clinical diagnostic microbiology. *FEMS Microbiol. Rev.* 36 380–407. 10.1111/j.1574-6976.2011.00298.x 22092265

[B3] Didelot X. Bowden R. Wilson D. J. Peto T. E. A. Crook D. W. (2012). Transforming clinical microbiology with bacterial genome sequencing. *Nat. Rev. Genet.* 13 601–612. 10.1038/nrg3226 22868263 PMC5049685

[B4] Dieckmann R. Helmuth R. Erhard M. Malorny B. (2008). Rapid classification and identification of *Salmonellae* at the species and subspecies levels by whole-cell matrix-assisted laser desorption ionization-time of flight mass spectrometry. *Appl. Environ. Microbiol.* 74 7767–7778. 10.1128/AEM.01402-1408 18952875 PMC2607147

[B5] Fagerquist C. K. Garbus B. R. Williams K. E. Bates A. H. Boyle S. Harden L. A. (2009). Web-based software for rapid top-down proteomic identification of protein biomarkers, with implications for bacterial identification. *Appl. Environ. Microbiol.* 75 4341–4353. 10.1128/AEM.00079-79 19411427 PMC2704838

[B6] Fagerquist C. K. Miller W. G. Harden L. A. Bates A. H. Vensel W. H. Wang G. (2005). Genomic and proteomic identification of a DNA-binding protein used in the “fingerprinting” of *Campylobacter* species and strains by MALDI-TOF-MS protein biomarker analysis. *Anal. Chem.* 77 4897–4907. 10.1021/ac040193z 16053303

[B7] Freiwald A. Sauer S. (2009). Phylogenetic classification and identification of bacteria by mass spectrometry. *Nat. Protoc.* 4 732–742. 10.1038/nprot.2009.37 19390529

[B8] Ge B. Wang F. Sjölund-Karlsson M. McDermott P. F. (2013). Antimicrobial resistance in *Campylobacter*: susceptibility testing methods and resistance trends. *J. Microbiol. Methods* 95 57–67. 10.1016/j.mimet.2013.06.021 23827324

[B9] Giebel R. A. Fredenberg W. Sandrin T. R. (2008). Characterization of environmental isolates of *Enterococcus* spp. by matrix-assisted laser desorption/ionization time-of-flight mass spectrometry. *Water Res.* 42 931–940. 10.1016/j.watres.2007.09.005 17931682

[B10] Goldstein J. E. Zhang L. Borror C. M. Rago J. V. Sandrin T. R. (2013). Culture conditions and sample preparation methods affect spectrum quality and reproducibility during profiling of *Staphylococcus aureus* with matrix-assisted laser desorption/ionization time-of-flight mass spectrometry. *Lett. Appl. Microbiol.* 57 144–150. 10.1111/lam.12092 23617594

[B11] Gorman R. Adley C. C. (2004). An evaluation of five preservation techniques and conventional freezing temperatures of -20°C and -85°C for long-term preservation of *Campylobacter jejuni*. *Lett. Appl. Microbiol.* 38 306–310. 10.1111/j.1472-765X.2004.01490.x 15214730

[B12] Hrabák J. Chudáčková E. Walková R. (2013). Matrix-assisted laser desorption ionization–time of flight (maldi-tof) mass spectrometry for detection of antibiotic resistance mechanisms: from research to routine diagnosis. *Clin. Microbiol. Rev.* 26 103–114. 10.1128/CMR.00058-12 23297261 PMC3553667

[B13] Iovine N. M. (2013). Resistance mechanisms in *Campylobacter jejuni*. *Virulence* 4 230–240. 10.4161/viru.23753 23406779 PMC3711981

[B14] Jolley K. A. Maiden M. C. (2010). BIGSdb: scalable analysis of bacterial genome variation at the population level. *BMC Bioinformatics* 11:595 10.1186/1471-2105-11-595 PMC3004885 21143983

[B15] Kaakoush N. O. Castaño-Rodríguez N. Mitchell H. M. Man S. M. (2015). Global epidemiology of *Campylobacter* infection. *Clin. Microbiol. Rev.* 28 687–720. 10.1128/CMR.00006-15 26062576 PMC4462680

[B16] Kliem M. Sauer S. (2012). The essence on mass spectrometry based microbial diagnostics. *Curr. Opin. Microbiol.* 15 397–402. 10.1016/j.mib.2012.02.006 22410108

[B17] Kostrzewa M. Sparbier K. Maier T. Schubert S. (2013). MALDI-TOF MS: an upcoming tool for rapid detection of antibiotic resistance in microorganisms. *Proteomics Clin. Appl.* 7 767–778. 10.1002/prca.201300042 24123965

[B18] Koubek J. Uhlik O. Jecna K. Junkova P. Vrkoslavova J. Lipov J. (2012). Whole-cell MALDI-TOF: rapid screening method in environmental microbiology. *Int. Biodeterior. Biodegrad.* 69 82–86. 10.1016/j.ibiod.2011.12.007

[B19] Lartigue M.-F. (2013). Matrix-assisted laser desorption ionization time-of-flight mass spectrometry for bacterial strain characterization. *Infect. Genet. Evol.* 13 230–235. 10.1016/j.meegid.2012.10.012 23159555

[B20] Lastovica A. J. (2006). Emerging *Campylobacter* spp.: the tip of the iceberg. *Clin. Microbiol. Newsl.* 28 49–56. 10.1016/j.clinmicnews.2006.03.004

[B21] Lastovica A. J. On S. L. W. Zhang L. (2014). “The Family Campylobacteraceae,” in *The Prokaryotes*, eds Rosenberg E. DeLong E. F. Lory S. Stackebrandt E. Thompson F. (Heidelberg: Springer), 307–335.

[B22] Laxminarayan R. Duse A. Wattal C. Zaidi A. K. M. Wertheim H. F. L. Sumpradit N. (2013). Antibiotic resistance—the need for global solutions. *Lancet Infect. Dis.* 13 1057–1098. 10.1016/S1473-3099(13)70318-9 24252483

[B23] Luangtongkum T. Jeon B. Han J. Plummer P. Logue C. M. Zhang Q. (2009). Antibiotic resistance in *Campylobacter*: emergence, transmission and persistence. *Future Microbiol.* 4 189–200. 10.2217/17460913.4.2.189 19257846 PMC2691575

[B24] Maloy S. Atlas R. M. (2014). *One Health**: People, Animals, and The Environment* Washington, DC: American Society of Microbiology 10.1128/9781555818432

[B25] Mitchell M. Mali S. King C. C. Bark S. J. (2015). Enhancing MALDI time-of-flight mass spectrometer performance through spectrum averaging. *PLoS ONE* 10:e0120932 10.1371/journal.pone.0120932 PMC4370844 25798583

[B26] Montgomery D. C. (2012). *Design and Analysis of Experiments*, 8th Edn Hoboken, NJ: John Wiley.

[B27] Payot S. Bolla J.-M. Corcoran D. Fanning S. Mégraud F. Zhang Q. (2006). Mechanisms of fluoroquinolone and macrolide resistance in *Campylobacter* spp. *Microbes Infect.* 8 1967–1971. 10.1016/j.micinf.2005.12.032 16713726

[B28] Pulido M. R. García-Quintanilla M. Martín-Peña R. Cisneros J. M. McConnell M. J. (2013). Progress on the development of rapid methods for antimicrobial susceptibility testing. *J. Antimicrob. Chemother.* 68 2710–2717. 10.1093/jac/dkt253 23818283

[B29] Randall L. P. Lemma F. Koylass M. Rogers J. Ayling R. D. Worth D. (2015). Evaluation of MALDI-ToF as a method for the identification of bacteria in the veterinary diagnostic laboratory. *Res. Vet. Sci.* 101 42–49. 10.1016/j.rvsc.2015.05.018 26267088

[B30] Sandrin T. R. Goldstein J. E. Schumaker S. (2013). MALDI TOF MS profiling of bacteria at the strain level: a review. *Mass Spectrom. Rev.* 32 188–217. 10.1002/mas.21359 22996584

[B31] Sauer S. Kliem M. (2010). Mass spectrometry tools for the classification and identification of bacteria. *Nat. Rev. Microbiol.* 8 74–82. 10.1038/nrmicro2243 20010952

[B32] Schmidt F. Fiege T. Hustoft H. K. Kneist S. Thiede B. (2009). Shotgun mass mapping of *Lactobacillus* species and subspecies from caries related isolates by MALDI-MS. *Proteomics* 9 1994–2003. 10.1002/pmic.200701028 19260002

[B33] Schubert S. Kostrzewa M. (2015). “Chapter 14 - MALDI-TOF mass spectrometry in the clinical microbiology laboratory; beyond identification,” in *Methods in Microbiology Vol. 42 Current and Emerging Technologies for the Diagnosis of Microbial Infections*, eds Sails A. Tang Y.-W. (Cambridge, MA: Academic Press), 501–524.

[B34] Schumaker S. Borror C. M. Sandrin T. R. (2012). Automating data acquisition affects mass spectrum quality and reproducibility during bacterial profiling using an intact cell sample preparation method with matrix-assisted laser desorption/ionization time-of-flight mass spectrometry. *Rapid Commun. Mass Spectrom.* 26 243–253. 10.1002/rcm.5309 22223309

[B35] Sparbier K. Schubert S. Weller U. Boogen C. Kostrzewa M. (2012). Matrix-assisted laser desorption ionization–time of flight mass spectrometry-based functional assay for rapid detection of resistance against β-lactam antibiotics. *J. Clin. Microbiol.* 50 927–937. 10.1128/JCM.05737-5711 22205812 PMC3295133

[B36] Wagenaar J. A. Newell D. G. Kalupahana R. S. Mughini-Gras L. (2015). “*Campylobacter*: animal reservoirs, human infections, and options for control,” in *Zoonoses - Infections Affecting Humans and Animals*, ed. Sing A. (Dordrecht: Springer), 159–177.

[B37] Wang Y. Huang W. M. Taylor D. E. (1993). Cloning and nucleotide sequence of the *Campylobacter jejuni* gyrA gene and characterization of quinolone resistance mutations. *Antimicrob. Agents Chemother.* 37 457–463. 10.1128/AAC.37.3.457 8384814 PMC187693

[B38] Welker M. (2011). Proteomics for routine identification of microorganisms. *Proteomics* 11 3143–3153. 10.1002/pmic.201100049 21726051

[B39] Welker M. Moore E. R. B. (2011). Applications of whole-cell matrix-assisted laser-desorption/ionization time-of-flight mass spectrometry in systematic microbiology. *Syst. Appl. Microbiol.* 34 2–11. 10.1016/j.syapm.2010.11.013 21288677

[B40] Wieczorek K. Osek J. (2013). Antimicrobial resistance mechanisms among *Campylobacter*. *BioMed Res. Int.* 2013:e340605 10.1155/2013/340605 PMC3707206 23865047

[B41] Wieser A. Schneider L. Jung J. Schubert S. (2011). MALDI-TOF MS in microbiological diagnostics—identification of microorganisms and beyond (mini review). *Appl. Microbiol. Biotechnol.* 93 965–974. 10.1007/s00253-011-3783-3784 22198716

[B42] World Health Organization [WHO] (2013). *The Global View of Campylobacteriosis.* Available at: http://www.who.int/foodsafety/publications/campylobacteriosis/en/

[B43] Zankari E. Hasman H. Cosentino S. Vestergaard M. Rasmussen S. Lund O. (2012). Identification of acquired antimicrobial resistance genes. *J. Antimicrob. Chemother.* 67 2640–2644. 10.1093/jac/dks261 22782487 PMC3468078

[B44] Zautner A. E. Masanta W. O. Tareen A. M. Weig M. Lugert R. Groß U. (2013). Discrimination of multilocus sequence typing-based *Campylobacter jejuni* subgroups by MALDI-TOF mass spectrometry. *BMC Microbiol.* 13:247 10.1186/1471-2180-13-247 PMC4228279 24195572

[B45] Zautner A. E. Masanta W. O. Weig M. Groß U. Bader O. (2015). Mass spectrometry-based PhyloProteomics (MSPP): a novel microbial typing method. *Sci. Rep.* 5:13431 10.1038/srep13431 PMC4548220 26303099

[B46] Zhang L. Borror C. M. Sandrin T. R. (2014). A designed experiments approach to optimization of automated data acquisition during characterization of bacteria with MALDI-TOF mass spectrometry. *PLoS ONE* 9:e92720 10.1371/journal.pone.0092720 PMC3963954 24662978

[B47] Zhao S. Tyson G. H. Chen Y. Li C. Mukherjee S. Young S. (2016). Whole-genome sequencing analysis accurately predicts antimicrobial resistance phenotypes in *Campylobacter* spp. *Appl. Environ. Microbiol.* 82 459–466. 10.1128/AEM.02873-15 26519386 PMC4711122

